# Development and Validation of the Short-LIMOS for the Acute Stroke Unit—A Short Version of the Lucerne ICF-Based Multidisciplinary Observation Scale

**DOI:** 10.3389/fresc.2022.857955

**Published:** 2022-04-05

**Authors:** Beatrice Ottiger, Tim Vanbellingen, Dario Cazzoli, Thomas Nyffeler, Janne M. Veerbeek

**Affiliations:** ^1^Neurocenter, Luzerner Kantonsspital, Lucerne, Switzerland; ^2^ARTORG Center for Biomedical Engineering Research, Gerontechnology and Rehabilitation Group, University Bern, Bern, Switzerland; ^3^Department of Psychology, University of Bern, Bern, Switzerland; ^4^Department of Neurology, Inselspital, Bern University Hospital, University of Bern, Bern, Switzerland

**Keywords:** stroke, acute, ADL, assessment, short-LIMOS, reliability, validation, ICF

## Abstract

**Introduction:**

At hospital stroke units, the time available to assess the patient's limitations in activities and participation is limited, although being essential for discharge planning. Till date, there is no quick-to-perform instrument available that captures the patient's actual performance during daily activities from a motor, cognitive, and communication perspective within the International Classification of Functioning, Disability and Health (ICF) framework. Therefore, the aim was to develop and validate a shortened version of the Lucerne ICF-Based Multidisciplinary Observation Scale (Short-LIMOS) that observes the patient's performance across ICF-domains and is applicable in the context of an acute stroke unit.

**Methods:**

The Short-LIMOS was developed by reducing the original 45-item LIMOS to the ten most important items using a multivariable linear regression ANOVA with data of 836 stroke patients collected during inpatient neurorehabilitation. The Short-LIMOS's reliability, validity, and responsiveness were evaluated with data of 416 stroke patients in the acute stroke unit.

**Results:**

A significant equation [*F*_(10,825)_ = 232.083] with *R*^2^ of 0.738 was found for the following ten items for the Short-LIMOS: maintaining a body position (d415), changing basic body position (d410), climbing stairs (d4551), eating (d550), dressing (d540), communicating with—receiving—written messages (reading) (d325), applying knowledge, remembering facts (d179), solving complex problems (d1751), making simple decisions (d177), and undertaking a simple task (d2100). Principal component analysis revealed a Short-LIMOS motor and a Short-LIMOS cognition/communication component. The Short-LIMOS had a high internal consistency and good test-retest reliability. A moderate construct validity was shown by the significant correlation with the Barthel Index. The Short-LIMOS had neither floor nor ceiling effects.

**Discussion and Conclusion:**

The developed Short-LIMOS was found to be reliable and valid within a population of (hyper)acute and subacute stroke patients. The added value of this multidisciplinary assessment is its comprehensiveness by capturing the patient's actual performance on the motor, cognitive, and communication domain embedded in an ICF-framework in <10 mins.

## Introduction

In an acute stroke unit setting, impairments due to stroke need to be detected within a noticeably short time in order to make appropriate decisions on acute medical treatment. In addition to acute medical treatment, it is crucial to assess which activities of daily living (ADLs) the patient can still perform and which not, as the ability to perform ADLs has been shown to be an important factor to consider in discharge planning (1–3). This requires an assessment tool that can capture and measure the patient's actual ADL performance. An acute stroke unit setting is not optimally suited for capturing the patients' actual ADL performance level: the premises are usually oriented toward acute medical care, and monitoring facilities often prevent patients to perform certain ADLs by themselves, even though they might theoretically be able to do so. Therefore, the observation and reliable assessment of ADL performance on acute stroke units represents a challenge. In this context, the development and validation of a specific ADL assessment tool for the acute stroke unit seems to be of crucial importance.

The Lucerne ICF-based Multidisciplinary Observation Scale (LIMOS) was developed to assess stroke patients' performance in the ADLs across the domains of the International Classification of Functioning, Disability and Health (ICF) (4). The LIMOS has an adequate reliability (4), validity (4, 5), and responsiveness (6), and is highly predictive of the patients' discharge destination after inpatient stroke rehabilitation (3). A central strength of the LIMOS is its comprehensiveness, i.e., it does not only include physical function during the ADLs, but also items regarding communication, learning and applying knowledge, interpersonal relationships, general tasks, and domestic life. Another advantage of the LIMOS is that—in contrast to, e.g., the Functional Independence Measure (FIM) (7) or the Barthel Index (BI) (8)—it does not present floor or ceiling effects (9, 10). Therefore, the LIMOS is particularly suited for the use in a subacute, multidisciplinary neurorehabilitation setting. However, with its 45 items, the scale is too extensive for the use in an acute stroke unit setting, in which decisions, amongst others regarding discharge destination, must be made in a noticeably short amount of time. Thus, for the acute stroke unit setting, a shortened version of the LIMOS is required. Apart from a short administration time, adequate measurement properties of the scale are needed to be useful in the environment of an acute stroke unit. Measurement properties reflect the quality of an outcome measure and can be assigned to one of the following three domains: reliability, validity, and responsiveness (11). Reliability is defined as “the degree to which the measurement is free from measurement error” (11). Validity reflects “the degree to which a health-related patient-reported outcome instrument [or: outcome measure] measures the construct(s) it purports to measure” (11). Finally, responsiveness refers to “the ability of a health-related patient-reported outcome instrument [or: outcome measure] to detect change over time in the construct to be measured” (11).

The aim of the present study was to develop a short version of the LIMOS (so-called Short-LIMOS), and to investigate its reliability, validity, and responsiveness. The goal of the Short-LIMOS was to be suitable for the use in an acute stroke unit setting, and to reflect not only the patients' physical functioning, but also their cognitive and communication performance.

## Materials and Methods

The development and validation of the Short-LIMOS was achieved in two phases. In the first phase, the Short-LIMOS was developed by reducing the original 45-item LIMOS to the ten most important items. In the second phase, the Short-LIMOS's reliability, validity, responsiveness, and floor and ceiling effects were assessed.

### Phase 1: Development of the Short-LIMOS

#### Study Design

A retrospective analysis of data routinely collected during inpatient stroke rehabilitation at the Neurocenter of the Luzerner Kantonsspital, Switzerland, between January 2014 and February 2019, was conducted.

#### Participants

Data from 836 stroke patients were analyzed. Patients with a cerebral stroke of any type, as confirmed by brain computed tomography or magnetic resonance imaging, were included. Patients were not included in the study if they were admitted for re-rehabilitation beyond the early subacute phase post-stroke (12). Patients received multidisciplinary rehabilitation according to national guidelines and local protocols (13), in which the focus lays on repetitive, task-oriented training.

#### Data Collection

##### Socio-Demographic Data

Medical and demographic data such as age, sex, diagnosis, time since stroke, and length of stay in the neurorehabilitation center were collected from patient records.

##### Lucerne ICF-Based Multidisciplinary Observation Scale

With the LIMOS, the stroke patients' performance during the ADL is assessed in a reliable and valid manner (4, 6). The LIMOS was administered as part of the clinical routine by trained members of the multidisciplinary team (i.e., nurses, occupational therapists, physical therapists, speech therapists, and neurologists). Administration occurred within the first 72 h after admission to the neurorehabilitation center, and again within the last 72 h before discharge. The LIMOS is based on the ICF framework (14), and consist of 45 items that are categorized in four components: interpersonal activities, motor and self-care; communication; knowledge and general tasks; and domestic life. The patients are observed during everyday hospital life and not in a test situation; this is important since the patients' behavior can be influenced by the test situation itself (i.e., induce the patients to overachieve and not to behave at their typical level of performance). For each item, the level of assistance needed by the patient is assessed by the respective health professional by means of a 5-point Likert scale, with higher scores reflecting a higher independence degree, i.e., 1 = the patient is not able to fulfill a task at all or needs more than 75% of assistance (i.e., complete assistance); 2 = the patient is able to fulfill a task with an assistance of 25–75% (i.e., severe assistance); 3 = the patient is able to fulfill a task with an assistance of <25%, or under supervision (i.e., moderate assistance); 4 = the patient is able to fulfill a task independently, but needs increased time and/ or auxiliary materials/ aids (i.e., slight assistance); and, 5 = the patient is able to fulfill a task independently (i.e., no assistance needed). Correspondingly, the LIMOS total score (calculated as the sum of all item scores) ranges from 45 to 225, with higher scores representing a higher degree of independence (4, 6).

#### Statistical Analyses

For all analyses, a two-tailed *p*-value with a critical threshold of <0.05 was used for accepting statistical significance. Descriptive statistics for nominal data were expressed as number of patients and percentages. Descriptive statistics for non-normally distributed quantitative data included median as well as first and third quartiles (Q1–Q3). Descriptive statistics for normally distributed data included mean and standard deviation.

In order to develop the Short-LIMOS, we aimed to define the LIMOS items that, at admission to the neurorehabilitation center (independent variables, see Supplementary Material 1), could explain most of the ADL performance at discharge, as assessed by the LIMOS total score (dependent variable). For this purpose, we first performed a multivariable, stepwise, forward linear regression analysis ANOVA, followed by an enter method linear regression ANOVA.

### Phase 2: Determine Measurement Properties Short-LIMOS

#### Study Design

A prospective analysis of data collected between April 2019 and March 2021 as part of the clinical routine at the acute stroke unit at the Neurocenter of the Luzerner Kantonsspital, Switzerland was conducted.

#### Participants

Acute stroke patients were tested in the acute stroke unit. Patients received multidisciplinary rehabilitation according to national guidelines and local protocols (13), in which the focus lays on repetitive, task-oriented training in the acute stroke unit.

#### Data Collection

##### Socio-Demographic Data

Medical and demographic data such as age, gender, diagnosis, time since stroke, National Institutes of Health Stroke Scale (NIHSS) (15, 16) score, and length of stay in the acute stroke unit were collected from patient records.

##### Short-LIMOS

Each stroke patient was assessed with the Short-LIMOS within 72 h from admission to the acute stroke unit by trained occupational and physical therapists. These therapists were familiar with the use of the LIMOS, because they have been working at the neurorehabilitation center as well as at the acute stroke unit of the same institution. After a presentation and workshop about the development of the Short-LIMOS with the occupational and physical therapy teams, the use of the Short-LIMOS was introduced at the acute stroke unit. The assessment of the Short-LIMOS is provided in the Appendix (see Supplementary Material 2).

##### Barthel Index

The BI measures ten items of basic everyday functions in self-care and mobility (8). The total score ranges from 0 to 20, with a score of 20 points indicating full independency in ADLs (17). The BI is recorded as part of the clinical routine within the first 24 h after admission by the nurses of the acute stroke unit.

#### Statistical Analyses

For all analyses, a two-tailed *p* value with a critical threshold of <0.05 was used for accepting statistical significance. Descriptive statistics for nominal data were expressed as number of patients and percentages. Descriptive statistics for non-normally distributed quantitative data included median as well as first and third quartiles (Q1–Q3). Descriptive statistics for normally distributed data included mean and standard deviation.

#### Reliability

##### Internal Consistency

Cronbach's alpha was calculated for testing the degree of interrelatedness among items [i.e., internal consistency (11)] of the Short-LIMOS. In addition, Cronbach's alpha was calculated for each common dimension that resulted from the principal component analysis (PCA). A value above 0.70 was considered to reflect an acceptable homogeneity among items within the total scale (11, 18).

##### Test–Retest Reliability

Test-retest reliability is “the extent to which scores for patients who have not changed are the same for repeated measurement under several conditions: for example, using different sets of items over time (test-retest)” (11). To determine this measurement property, data from patients transferred from the acute stroke unit to the neurorehabilitation center were used (**Figure 2**). For this purpose, the ten LIMOS items that were included in the Short-LIMOS at admission to the neurorehabilitation center were compared with the ones of the Short-LIMOS performed at the acute stroke unit. For this comparison, the Intraclass Correlation Coefficient (ICC: two-way random model with effect of absolute agreement) (19) was calculated in a subgroup of 85 patients. An ICC value above 0.70 was considered as acceptable (20).

##### Measurement Error

Measurement error is defined as “the systematic and random error of a patient's score that is not attributed to true changes in the construct to be measured” (11). To assess the measurement error, data from patients transferred from the acute stroke unit to the neurorehabilitation center were analyzed (**Figure 2**). The measurement error was determined by calculating the standard error of measurement (SEM), which is a measure of the error in the scores that is not due to true changes. The SEM was calculated by dividing the standard deviation of the difference between test and retest scores by square root of two $\left(SDdiff\right)/\sqrt{2}$ (21, 22).

#### Validity

##### Structural Validity

Structural validity refers to “the degree to which the scores of a health-related patient reported instrument [or: outcome measure] are an adequate reflection of the dimensionality of the construct to be measured” (11). A PCA was performed to explore the underlying dimensions of the Short-LIMOS. With the PCA, the degree to which the ten items of the Short-LIMOS were most strongly correlated with each other was defined, which allowed to build common dimensions within the total observation scale. A minimum eigenvalue of 1 was specified as extraction criterion. The criterion for factor loading was set at 0.40 (11, 23).

##### Hypothesis Testing for Construct Validity

Hypotheses testing refers to construct validity and is defined as “the degree to which the scores of a health-related patient reported instrument [or: outcome measure] are consistent with hypotheses (for instance with regard to internal relationships, relationships to scores of other instruments, or differences between relevant groups) based on the assumption that the health-related patient reported instrument [or: outcome measure] validly measures the construct to be measured” (11). To strengthen construct validity, the Short-LIMOS scores were compared with the ones of the BI. We hypothesized that the sum score of the Short-LIMOS would positively and strongly correlate (i.e., *r*_*s*_ ≥ 0.60) with the total score of the BI. Due to the implementation of a new electronic patient record system in September 2019, the BI from this time point onwards could be extracted. Therefore, we calculated the Pearson's correlation in a subgroup of 278 patients. Correlation coefficients of < 0.25 indicate little or no relationship; 0.25–0.50 suggest a fair degree; values of 0.50–0.75 are moderate to good; and values above 0.75 are considered good to excellent (24).

##### Criterion Validity

To assess “the degree to which the scores of a health-related patient-reported outcome [or: outcome measure] are an adequate reflection of a ‘gold standard”’ (11), the Spearman Rank correlation coefficient between the LIMOS and Short-LIMOS was calculated.

#### Responsiveness

Based on the SEM, the distribution-based responsiveness was determined by calculating the Minimum Detectable Change $\left(MDC_{90}=SEM\;*\;1.65\;*\;\sqrt{2}\right)$, which describes with 90% certainty the amount of true change in subject status beyond measurement error (25). A Bland-Altman plot analysis was used to display the within-subject variability as well as the systematic difference between the Short-LIMOS items at acute stroke unit and the same ten items at admission to the neurorehabilitation center. The bias (mean difference; MD), its standard deviation (SD), as well as the upper and lower limits of agreement (defined as *MD* ± 1.96 * *SD*) were calculated (26).

#### Floor and Ceiling Effects

The floor and ceiling effects reflect the extent to which scores cluster at the bottom or at the top of the scale range, respectively. Floor and ceiling effects were considered as present if 15% of the respondents scored the lowest or highest score on a scale, respectively (27).

## Results

### Phase 1: Development of the Short-LIMOS

**Figure 1:**
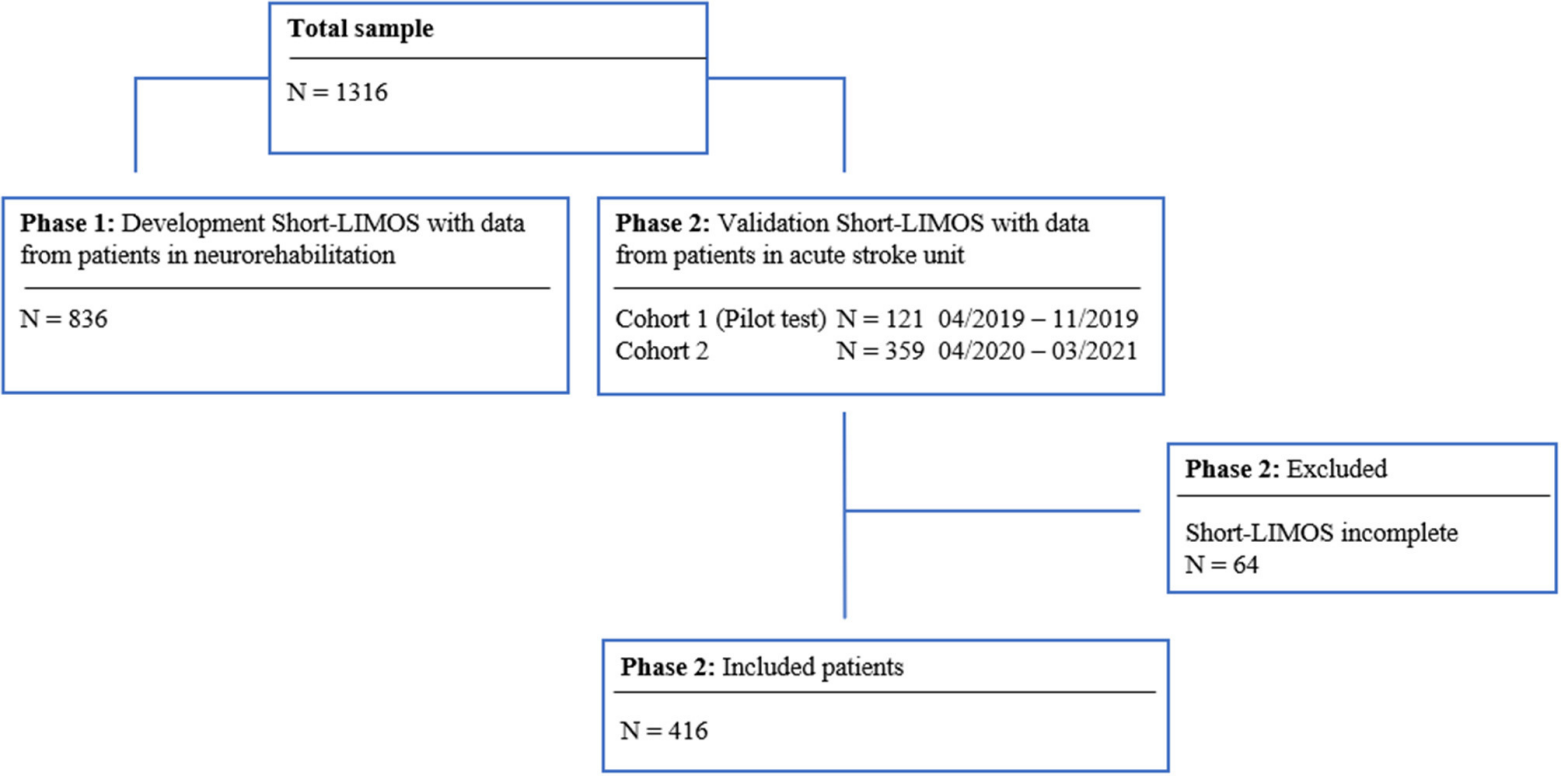
Flow chart of the patient sample for the development and validation phase.

The patient flow for the first phase is displayed in Figure 1. Baseline characteristics of the patients included are summarized in Table 1.

**Table 1:** Characteristics of the patient's study sample in Phase 1 and Phase 2.

**Characteristic**	**Phase 1**	**Phase 2**
		**Whole cohort**	**Subgroups**	
	**(*****N*** **=** **836)**	**Validation (*****N*** **=** **416)**	**Reliability (*****N*** **=** **83)**	**Construct validity (*****N*** **=** **278)**
Age, years^*^	71 (61–80)	73 (61–82)	70 (61–79)	72.5 (60–82)
Gender, female^**^	380 (45.5)	190 (46)	45 (54)	109 (39)
Stroke type, ischemic^**^	611 (73)/225 (27)	384 (92)/32 (8)	75 (90)/8 (10)	261 (94)/17 (6)
First-ever stroke, yes^**^	N/A	364 (87.5)	73 (88)	238 (85.6)
Time poststroke, days^*^	9 (7–14)	1 (1–2)	2 (1–3)	1 (1–2)
Length of stay acute stroke unit^*^	N/A	4 (2–7)	4 (4–5)	4 (2–7)
Length of stay rehabilitation center^*^	31 (20–46)	N/A	27 (18–50)	N/A
NIHSS admission acute stroke unit^*^	N/A	3 (1–7)	4 (3–8)	3 (1–3)
Thrombolysis, yes^**^	N/A	100 (24)	21 (25.3)	73 (26.3)
Thrombectomy, yes^**^	N/A	54 (13)	7 (8.4)	40 (14.4)
LIMOS admission rehabilitation center	128.96 (91.5–162.5)	N/A	133.4 (108.8–167.7)	N/A
LIMOS discharge rehabilitation center	170.5 (132.3–193.5)	N/A	177.4 (151–196.3)	N/A
Short-LIMOS admission acute stroke unit	N/A	33.71 (22.9–41.1)	30.7 (24.4–37.2)	33.75 (22.8–41.7)
Short-LIMOS admission neurorehabilitation	N/A	N/A	31.7 (25.4–39.7)	N/A
BI admission acute stroke unit	N/A	N/A	N/A	30 (20–50)

* *Median (quartile 1 – quartile 3)*;

** *N (%); BI, Barthel Index; LIMOS, Lucerne ICF-based Multidisciplinary Observation Scale; N/A, not applicable; NIHSS, National Institutes of Health Stroke Scale (when thrombolysis and/ or thrombectomy was applied, after acute medical intervention); Short-LIMOS, Shortened Version of the Lucerne ICF-based Multidisciplinary Observation Scale*.

#### Regression Analysis

The first linear regression analysis ANOVA showed that 12 LIMOS items were influencing variables: dressing (d540), maintaining a body position (d415), communicating with—receiving—written messages (reading) (d325), applying knowledge, remembering facts (d179), solving complex problems (d1751), basic interpersonal interactions (d710), changing basic body position (d410), calculating (d172), climbing stairs (d4551), eating (d550), undertaking a simple task (d2100), making simple decisions (d177). A significant regression equation was found [*F*_(12,823)_ = 199.514, *p* < 0.000] with an R^2^ of 0.744. Two items were removed, as they were classified as misfits by the LIMOS scale in an earlier performed Rasch analysis of the LIMOS (5). These items were basic interpersonal interactions (d710) and calculating (d172). The subsequent second linear regression analysis with the inclusion of the remaining ten LIMOS items also resulted in a significant equation [*F*_(10,825)_ = 232.083, *p* < 0.000] with an R^2^ of 0.738.

### Phase 2: Determine Measurement Properties Short-LIMOS

A total of 480 patients were included in the second phase (Figure 1). During the pilot study, all Short-LIMOS files were complete. In the second phase, 64 (13.3%) Short-LIMOS files were not or partially filled out (Figure 1). Comparing patients with complete and incomplete files, we noted that there was no significant difference in terms of age (*p* > 0.5) and sex (*p* > 0.5). However, the 64 patients with missing data showed a significantly lower NIHSS score at hospital admission (*p* < 0.001) and a shorter length of stay at the acute stroke unit (*p* = 0.004) compared to those whose Short-LIMOS files were completed. For further statistical analyses, data of 416 patients with completed Short-LIMOS files were used.

#### Patient Characteristics

A total of 416 patients were included in the validation study. The demographics and the baseline characteristics of the patients are presented in Table 1.

#### Reliability

##### Internal Consistency

An excellent internal consistency was found for the whole Short-LIMOS (Cronbach's alpha = 0.959) and its motor (Cronbach's alpha 0.948) and cognition/communication (Cronbach's alpha 0.952) subscales.

##### Test-Retest Reliability

**Figure 2:**
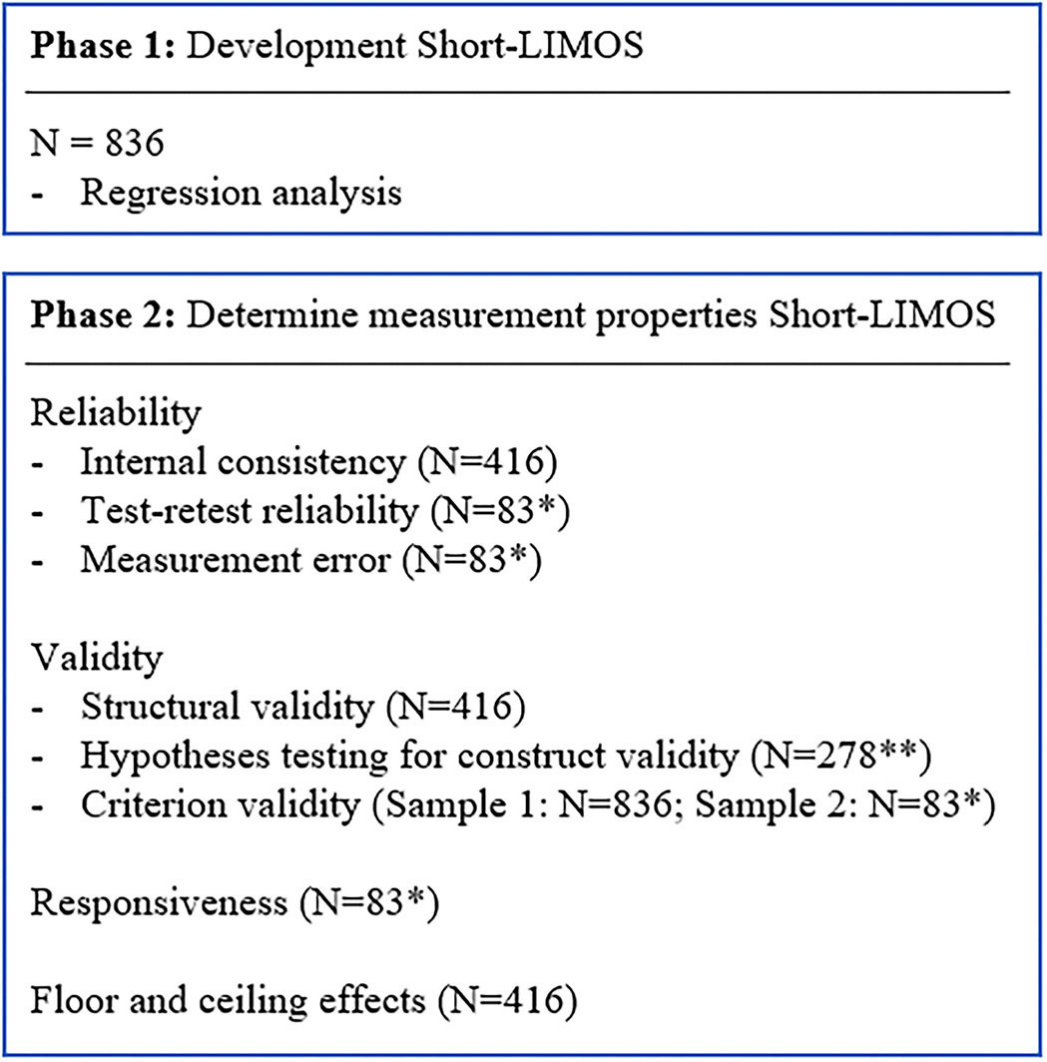
Flow chart of the statistical analyses for the development and validation phase. *Patients transferred from acute stroke unit to neurorehabilitation within 1–4 days; **Short-LIMOS total score and Barthel Index total score at admission acute stroke unit.

Eighty-three patients were transferred from the acute stroke unit to inpatient neurorehabilitation within 1–4 days after being assessed with the Short-LIMOS [median 3 (2–3) days] (Figure 2). The test-retest reliability of the Short-LIMOS within 4 days was excellent (ICC = 0.922; 95% confidence interval 0.856–0.955; *p* < 0.001).

##### Measurement Error

**Figure 3:**
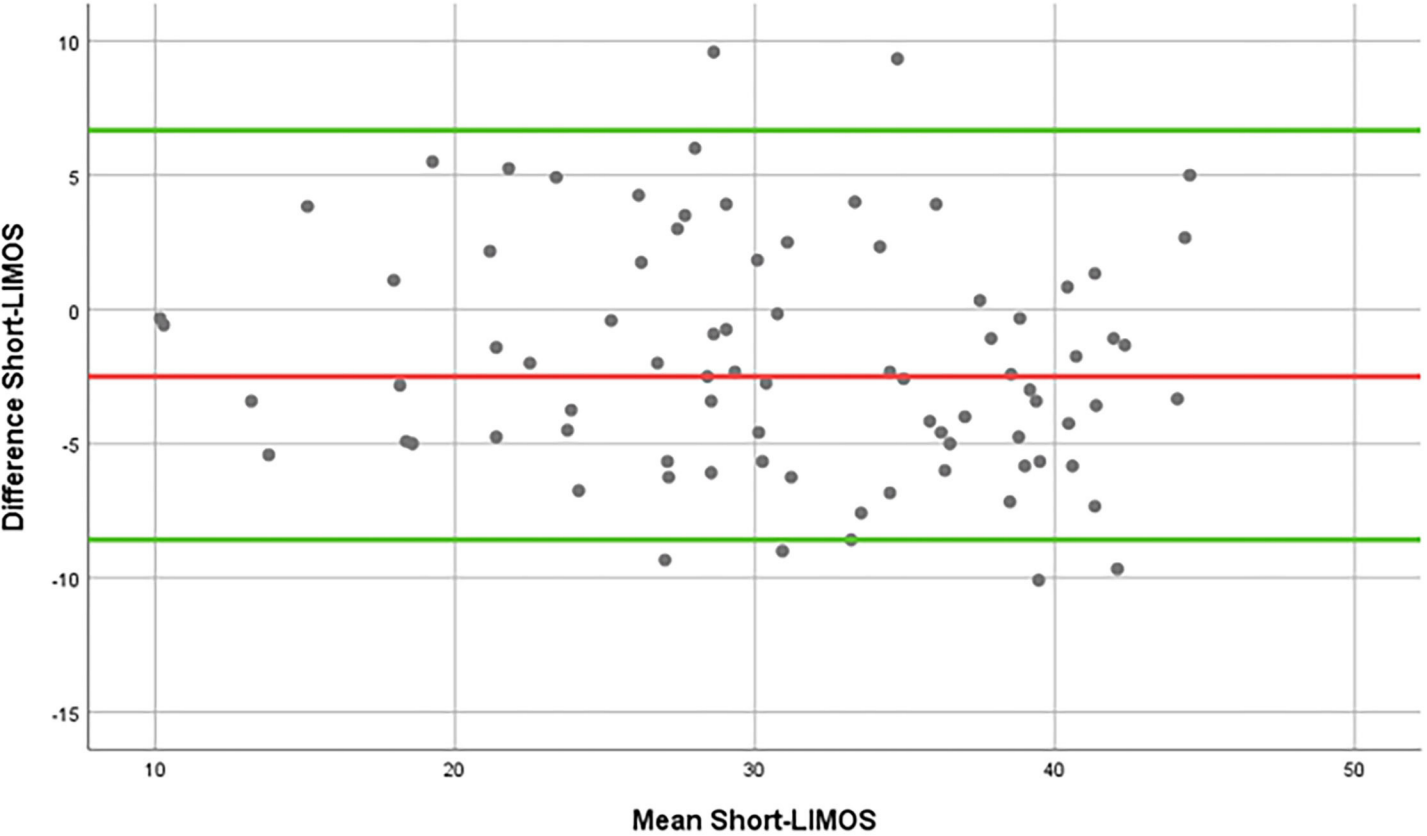
Bland-Altman plot of the Short-LIMOS.

The SEM was 3.09 points. The Bland–Altman plot showed the comparison between the two measurement time points (Figure 3).

#### Validity

##### Structural Validity

The PCA resulted in two components with an eigenvalue of above 1.0. A first, strong component had an eigenvalue of 7.41 and explained 74.12% of the variance and the second component had an eigenvalue of 1.09, which explained 10.91% of the variance. Both components together explained 85.03% of the variance.

The PCA revealed that all the ten items of the Short-LIMOS correlated at least with *r*_*s*_ = 0.3 with at least one other item, suggesting reasonable factorability. Secondly, the Kaiser-Meyer-Olkin measure of sampling adequacy was of 0.922, which is above the recommended value of 0.6, and Barlett's test of sphericity was significant (χ^2^_(5,190.25)_, *df* 45, *p* < 0.001). The diagonals of the anti-image correlation matrix were also all over 0.5. The rotated factor loadings of the two components are presented in Table 2.

**Table 2:** Principal component analysis of the Short-LIMOS.

**Item (ICF coding)**	**Component 1 (Short-LIMOS motor)**	**Component 2 (Short-LIMOS cognition/ communication)**
Maintaining a body position (d415)	0.847	
Changing basic body position (d410)	0.900	
Climbing stairs (d4551)	0.853	
Eating (d550)	0.851	
Dressing (d540)	0.721	
Communicating with—receiving—written messages (reading) (d325)		0.873
Solving complex problems (d1751)		0.822
Applying knowledge, remembering facts (d179)		0.880
Making simple decisions (d177)		0.846
Undertaking a simple task (d2100)		0.697

*Short-LIMOS, Shortened Version of the Lucerne ICF-based Multidisciplinary Observation Scale; ICF, International Classification of Functioning, Disability and Health*.

##### Hypothesis Testing for Construct Validity

The hypothesis concerning construct validity was supported by a moderate correlation above 0.60 (*r*_*s*_ = 0.67, *p* < 0.001) between Short-LIMOS total and BI total at admission to the acute stroke unit.

##### Criterion Validity

The comparison of the Short-LIMOS with the original LIMOS showed a high correlation of *r*_*s*_ = 0.99 in Sample 1 and *r*_*s*_ = 0.98 in Sample 2.

#### Responsiveness

The MDC_90_ was 7.22 points, which indicates a reliable change of 18.05% on the total range of 40 points (range from 10 to 50 points).

#### Floor and Ceiling Effects

With 3.4% of patients achieving the lowest score, and 1.7% achieving the highest score, no floor and ceiling effects were found for the total Short-LIMOS score.

## Discussion

The shortened version of the Lucerne ICF-Based Multidisciplinary Observation Scale (so called Short-LIMOS) developed in this study, for use at an acute stroke unit consists of 10 items that capture stroke patients' performance in daily activities from a motor, cognitive, and communication perspective. The Short-LIMOS was found to be reliable, valid, and responsive in the (hyper)acute phase (0 h to 7 days) (12) post-stroke.

The Short-LIMOS consists of two constructs, namely motor performance, and cognitive and communication performance, and structural validity reflects an adequate dimensionality of these two constructs. The test-retest reliability of the Short-LIMOS was excellent, meaning that the scale is stable by repeated measurement of patients who did not clinically change in their performance. This makes the test utmost suitable for repeated measures to track the patient's progress (i.e., follow-up). Based on the responsiveness, a change of 7.22 points can be interpreted with 90% confidence that the patient has indeed changed. The absence of floor and ceiling effects indicates that within the Short-LIMOS, there is room to detect improvement or deterioration at both ends of the scale.

The reduction in the number of the scale items from 45 to 10 resulted in an outcome measure that is feasible to perform in the highly dynamic acute stroke unit, at which the available time for assessments is limited. The items can be rated within one therapy session, making the administration time comparable to the one of the direct observations in the 10-item BI (8). However, in contrast to the BI, within this time window the short-LIMOS allows not only to assess the patients' motor performance in daily activities, but also their cognitive performance, including communication. Critically, this allows a more comprehensive assessment, which provides important information to determine the patients' treatment needs based on the identified impairments, but also for the planning of a suitable discharge destination. The correlation coefficient (*r*_*s*_) of 0.67 between the Short-LIMOS and the BI shows indeed that the Short-LIMOS is not merely a copy of this latter established test but provides additional information to the patient's motor function, namely the cognitive and communication abilities. Furthermore, the Short-LIMOS is a multidisciplinary observational scale, which has the advantage that none of the single disciplines is solely responsible for all observations, i.e., these can be divided across neurologists, nurses, occupational therapists, physical therapists, and speech therapists. This results in an administration burden of <5 mins per discipline.

The criterion validity showed a high correlation between the original LIMOS and its shortened version. This is not surprising, as the ten Short-LIMOS items were derived from the original LIMOS by means of a regression analysis and the corresponding R^2^ was 0.738. With the high correlation between the original and shortened LIMOS, one could raise the question whether both scales are needed? Our answer to this question is yes, as the target setting for each of the scales is different. The original LIMOS was developed for an inpatient neurorehabilitation setting (4) in which are more time and disciplines available to observe the patient's performance limitations during daily activities. In addition, the patients' situation allows a more extended examination when compared to the acute stroke unit setting. A more profound observation results in a more detailed profile of the patient's limitations, based on which focused rehabilitation goals can be defined and interventions be selected. Contrary to this, the newly developed Short-LIMOS's field of application is the stroke unit, where there is little time and space available to acquire a first objective assessment regarding the patient's performance with accompanying deficits.

Although we developed the Short-LIMOS for clinical purposes, this assessment could also be applied in acute stroke research projects to capture the patient's motor, cognitive and communication performance. The lack of floor and ceiling effects of the Short-LIMOS makes is utmost suitable for investigating changes over time, one point of criticism to which the BI is often subject to (28), in both clinical settings and research projects. Indeed, a sequential re-assessment of the Short-LIMOS would allow to profile the patients' progress over time, also beyond the acute phase. If a more detailed assessment is needed at later time points, then the full LIMOS (4) can be administered. A comparison with the Short-LIMOS scores would still be possible, as the present work shows that the two scales are highly correlated (*r*_s_ = 0.99).

## Limitations

For determining the test-retest reliability and measurement error of the Short-LIMOS, the sample needs to be clinically stable in the interim period (11). Therefore, only data from patients who were transferred from the acute stroke unit to the internal neurorehabilitation center within 1–4 days after being assessed with the Short-LIMOS on the acute stroke unit were analyzed. As neurological improvement is particularly sustained early after stroke (29), this may also result in a change in daily activity performance. Nevertheless, we found an excellent ICC in the test-retest reliability analysis. Two other measurement properties for reliability, namely the intrarater as well as the interrater reliability, were not assessed in the present work and should be further investigated. However, for the original LIMOS, the interrater reliability on item-level ranged from moderate to almost perfect agreement for most items and therefore, it can be assumed that this is also the case for its shortened version (4).

With a value of 1.7%, the ceiling effect of the Short-LIMOS was very low. This value might be slightly underestimated, as 13.33% of the patients were not considered in the analysis due to no or incomplete data on the Short-LIMOS. This subset of patients did not differ significantly from those with complete Short-LIMOS data in terms of age and sex. They were, however, less severely neurologically impaired (NIHSS) and had a shorter length of hospital stay.

## Conclusion

The Short-LIMOS allows a reliable, valid, responsive, and rapid multidisciplinary observation of stroke patients' motor, cognitive and communication performance in daily activities in the context of an acute stroke unit. With that, the scale could provide useful information for discharge planning after acute stroke unit stay and designing rehabilitation. Future work should study the predictive ability of the Short-LIMOS for, for example, discharge destination, to further increase the evidence-based application of this scale. Furthermore, capturing the recovery profile of the patient's actual performance in daily activities from a physical, cognitive and speech perspective in parallel—using the Short-LIMOS—would shed new light on stroke recovery.

## Data Availability Statement

The original contributions presented in the study are included in the article/Supplementary Material, further inquiries can be directed to the corresponding author.

## Ethics Statement

The studies involving human participants were reviewed and approved by Cantonal Ethics Committee Northwest and Central Switzerland (BASEC-ID 2017-00998). The patients/participants provided their written informed consent to participate in this study.

## Author Contributions

BO, TV, TN, and JV contributed to conception and design of the study. BO organized the database. BO and TV performed the statistical analysis. BO and JV drafted the manuscript. TN, TV, and DC critically revised the manuscript for important intellectual content. All authors contributed to manuscript revision, read, and approved the submitted version.

## Funding

This study was supported by the SNF Grant No. 32003B_196915.

## Conflict of Interest

The authors declare that the research was conducted in the absence of any commercial or financial relationships that could be construed as a potential conflict of interest.

## Publisher's Note

All claims expressed in this article are solely those of the authors and do not necessarily represent those of their affiliated organizations, or those of the publisher, the editors and the reviewers. Any product that may be evaluated in this article, or claim that may be made by its manufacturer, is not guaranteed or endorsed by the publisher.

## Abbreviations

ADL: activities of daily living
ANOVA: analysis of variance
BI: Barthel Index
CI: confidence interval
COSMIN: Consensus-based Standards for the Selection of Health Measurement Instruments
ICC: Intra-class Correlation Coefficient
ICF: International Classification of Functioning, Disability and Health
LIMOS: Lucerne ICF-based Multidisciplinary Observation Scale
LUKS: Luzerner Kantonsspital
MD: mean difference
MDC: minimum detectable change
NIHSS: National Institutes of Health Stroke Scale
PCA: principal component analysis
SD: standard deviation
SDdiff: standard deviation of the difference
SEM: standard error of measurement
Short-LIMOS: Shortened Version of the Lucerne ICF-based Multidisciplinary Observation Scale
SRM: standardized response means
Q1: first quartile
Q3: third quartile.
