# Supplementary material for: Development and Validation of the Short-LIMOS for the Acute Stroke Unit—A Short Version of the Lucerne ICF-Based Multidisciplinary Observation Scale
Source: Front Rehabil Sci. 2022 Apr 5;3:857955. doi: 10.3389/fresc.2022.857955 (PMC9397680; doi:10.3389/fresc.2022.857955)
Supplement: Supplementary file 1 [file Data_Sheet_1.PDF]

Supplementary Material 1 | Linear regression ANOVA with the 45 LIMOS items

	First linear regression, stepwise method, ANOVA, 45 items*					Second linear regression, enter method, ANOVA, 10 items*				
	Unstandardized coefficients β	Coefficients std. error	Standardized coefficients β	p-value	95% CI for β	Unstandardized coefficients β	Coefficients std. error	Standardized coefficients β	p-value	95% CI for β
1. Basic interpersonal interactions (d710)	3.421	.914	.096	.000	1.626 – 5.215					
2. Maintaining a body position (d415)	6.781	1.640	.191	.000	3.562 – 9.999	7.851	1.641	.221	.000	4.630 – 11.071
3. Changing basic body position (d410)	7.167	1.895	.202	.000	3.446 – 10.887	6.301	1.905	.178	.001	2.561 – 10.041
4. Transferring oneself (d4200)										
5. Lifting and carrying objects (d430)										
6. Fine hand use (d440)										
7. Hand and arm use (d445)										
8. Walking (d450)										
9. Walking long distances (d4501)										
10. Climbing stairs (d4551)	-3.073	1.209	-.095	.011	-5.446 – -.700	-3.361	1.218	-.104	.006	-5.751 – -.971
11. Moving around in another way (d455)										
12. Using a wheelchair (d465)										
13. Washing oneself (d510)										
14. Caring for body parts (d520)										
15. Toileting (d530)										
16. Dressing (d540)	6.132	1.413	.188	.000	3.358 – 8.905	6.858	1.415	.210	.000	4.080 – 9.637
17. Eating (d550)	-2.289	1.018	-.073	.025	-4.287 – -.291	-2.081	1.028	-.066	.000	-4.098 – -.063
18. Drinking (d560)										
19. Looking after one's health (d570)										
20. Coping at night (d598)										
21. Communication in general (d329)										
22. Communication with – receiving – spoken messages (d310/d315)										
23. Communicating with - receiving – written messages (d325)	3.665	.729	.126	.000	2.234 – 5.096	4.578	.701	.157	.000	3.201 – 5.955
24. Speaking (d330)										
25. Writing (d345)										
26. Acquiring basic skills (d1550)										

27. Acquiring complex skills (d1551)										
28. Focusing attention (d160)										
29. Thinking (d163)										
30. Solving simple problems (d1750)										
31. Solving complex problems (d1751)	3.720	1.225	.096	.002	1.315 – 6.125	3.779	1.234	.098	.002	1.357 – 6.201
32. Applying knowledge, remembering facts (d179)	4.148	1.176	.113	.000	1.839 – 6.456	4.895	1.178	.133	.000	2.584 – 7.207
33. Applying knowledge, orientation (d179)										
34. Applying knowledge, visual spatial perception (d179)										
35. Calculating (d172)	2.091	.888	.066	.019	.349 – 3.834					
36. Making simple decisions (d177)	1.866	1.159	.056	.108	-.410 – 4.141	3.340	1.109	.100	.003	1.163 – 5.517
37. Making complex decisions (d177)										
38. Undertaking a simple task (d2100)	2.856	1.309	.078	.029	.286 – 5.426	3.426	1.317	.094	.009	.841 – 6.011
39. Undertaking a complex task (d2101)										
40. Carrying out daily routine (d230)										
41. Acquisition of goods and services (d620)										
42. Preparing a simple meal (d6300)										
43. Preparing a complex meal (d6301)										
44. Doing housework (d640)										
45. Assisting others (d660)										

* only the significant items have been listed; ANOVA = Analysis of Variance; CI = Confidence Interval; LIMOS = Lucerne ICF-based Multidisciplinary Observation Scale; std = Standard; β = Beta.
