# Supplementary material for: Development and Validation of the Short-LIMOS for the Acute Stroke Unit—A Short Version of the Lucerne ICF-Based Multidisciplinary Observation Scale
Source: Front Rehabil Sci. 2022 Apr 5;3:857955. doi: 10.3389/fresc.2022.857955 (PMC9397680; doi:10.3389/fresc.2022.857955)
Supplement: Supplementary file 2 [file Data_Sheet_2.PDF]

Supplementary Material 2 | Short-LIMOS

Acute Stroke Unit

luzerner kantonsspital
LUZERN SURSEE WOLHUSEN

Patient
Name ____________________
Date of birth ____________________

Short - Lucerne ICF-Based Multidisciplinary Observation Scale (Short-LIMOS)

1 = patient is not able to fulfil a task or needs assistance more than 75% (corresponding to "complete")
2 = patient is able to fulfil tasks with assistance of 25% to 75% (corresponding to "severe")
3 = patient is able to fulfil tasks with assistance less than 25% or under supervision (corresponding to "moderate")
4 = patient is able to fulfil tasks independently but needs more time and/or with auxiliary materials/aids (corresponding to "slight")
5 = patient is able to fulfil tasks independently (corresponding to "none")

		Admission Date (to be filled out within 72 hours)
		«date»
**1**	**Maintaining a body position (d415)**	#DIV/0!
	Maintaining a lying position (d4150)	
	Maintaining a sitting position (d4153)	
	Maintaining a standing position (d4154)	
**2**	**Changing basic body position (d410)**	#DIV/0!
	Turning in bed (d4108)	
	Lying down (d4100) - From supine lying to sitting at the edge of bed and vice versa	
	Sitting (d4103) - Getting up and sitting down on a chair	
	Standing (d4104) - Lying down on the floor and getting up again	
**3**	**Climbing stairs (d4551)**	
**4**	**Dressing (d540)**	#DIV/0!
	Putting on clothes (d5400) and taking off clothes (d5401) one's upper body	
	Putting on clothes (d5400) and taking off clothes (d5401) one's lower body	
**5**	**Eating (d550)**	
**6**	**Communicating with - receiving - written messages (d325)**	#DIV/0!
	Understanding individual words	
	Understanding a simple text	
	Understanding sophisticated text	
**7**	**Solving complex problems (d1751)**	
**8**	**Applying knowledge, remembering facts (d179)**	
**9**	**Making simple decisions (d177)**	
**10**	**Undertaking a simple task (d2100)**	
	**Sum score Short-LIMOS (minimum 10 points to maximum 50 points)**	**#DIV/0!**
